# Investigation of Liver Injury of *Polygonum multiflorum* Thunb. in Rats by Metabolomics and Traditional Approaches

**DOI:** 10.3389/fphar.2017.00791

**Published:** 2017-11-01

**Authors:** Yun-Xia Li, Xiao-Hong Gong, Mei-Chen Liu, Cheng Peng, Peng Li, Yi-Tao Wang

**Affiliations:** ^1^State Key Laboratory of Quality Research in Chinese Medicine, Institute of Chinese Medical Sciences, University of Macau, Macau, China; ^2^State Key Laboratory Breeding Base of Systematic Research, Development and Utilization of Chinese Medicine Resources, Pharmacy College, Chengdu University of Traditional Chinese Medicine, Chengdu, China

**Keywords:** *Polygonum multiflorum* Thunb., toxicity, metabolomics, biochemical and histopathology examination, rat model

## Abstract

Liver injury induced by *Polygonum multiflorum* Thunb. (PM) have been reported since 2006, which aroused widespread concern. However, the toxicity mechanism of PM liver injury remained unclear. In this study, the mechanism of liver injury induced by different doses of PM after long-term administration was investigated in rats by metabolomics and traditional approaches. Rats were randomly divided into control group and PM groups. PM groups were oral administered PM of low (10 g/kg), medium (20 g/kg), high (40 g/kg) dose, while control group was administered distilled water. After 28 days of continuous administration, the serum biochemical indexes in the control and three PM groups were measured and the liver histopathology were analyzed. Also, UPLC-Q-TOF-MS with untargeted metabolomics was performed to identify the possible metabolites and pathway of liver injury caused by PM. Compared with the control group, the serum levels of ALT, AST, ALP, TG, and TBA in middle and high dose PM groups were significantly increased. And the serum contents of T-Bil, D-Bil, TC, TP were significantly decreased. However, there was no significant difference between the low dose group of PM and the control group except serum AST, TG, T-Bil, and D-Bil. Nine biomarkers were identified based on biomarkers analysis. And the pathway analysis indicated that fat metabolism, amino acid metabolism and bile acid metabolism were involved in PM liver injury. Based on the biomarker pathway analysis, PM changed the lipid metabolism, amino acid metabolism and bile acid metabolism and excretion in a dose-dependent manner which was related to the mechanism of liver injury.

## Introduction

*Polygonum multiflorum* (PM), the root of *Polygonum multiflorum* Thunb., has been applied in clinic for more than a thousand years with remarkable efficacy as nourishing Chinese medicine (Lin L. et al., 2015; Zhang et al., 2015). The long application history in China rarely reported PM toxicity in ancient herbal works. But in recent years, Medicines and Healthcare Products Regulatory Agency in British and Adverse Drug Reactions Advisory Committee in Australia have released the warning message in liver injury of PM since 2006 (Cárdenas et al., 2006; Cho et al., 2009; Wu et al., 2012; Dong et al., 2014). China Drug and Food Administration issued a warning message of liver injury with PM preparations in 2013 (Lian et al., 2013; Miao and Yun, 2013). Since then, more than 100 cases of liver damage were reported which has attracted worldwide attention to PM safety as a commonly used traditional Chinese medicine (Teschke et al., 2014; Lei et al., 2015). Therefore, it is necessary to clarify the related mechanism of liver injury caused by PM and provide scientific basis for clinical rational drug use.

Metabolomics study the species, quantity and changes of organism endogenous metabolites. Through a comprehensive and systematic investigation of the metabolic profiles change, metabolomics analyze the difference of metabolic fingerprints and obtain the corresponding biomarkers combined with pattern recognition and other chemical information technology (Bose et al., 2014; Li et al., 2014; Su et al., 2014). Then qualitative analysis and quantitative determination of biomarkers are carried out to reveal the relationship and dynamic laws between physiological and pathological changes (Ma et al., 2016; Zhang et al., 2016b).

Some metabolomics studies have been carried out to investigate the HSW toxicity. A targeting quantitative metabolomics reported the effect of PM on liver damage concentrating on nine kinds of bile acid changes (Dong et al., 2015). A study investigated the effect of different extract of PM on idiosyncratic drug-induced liver injury (Li et al., 2016), and another urine metabolomics study evaluated the liver toxicity of PM extract by 75% ethanol–water (Zhang et al., 2016a). As we know, traditional Chinese medicine is always decocted with water in clinical application and the dosage is also a key factor influencing the herb toxicity. However, no related report can be found in investigating the impairment level of different PM dosage on liver damage. And most reported study used the HSW extract by organic solvent which was not in accordance with the actual clinical application.

Therefore, serum samples from rats with liver injury were systematically investigated using traditional biochemical analysis, pathological observation and non-targeted metabolomics method to evaluate the relationship between the dosage of PM and liver damage. The metabolic profiles of serum of rats with liver injury and normal rats were analyzed to screen serum biomarkers of metabolic disorders caused by liver damage. The metabolic pathways were explored by combining the biochemical indexes and metabolic markers to clarify liver injury mechanism of PM.

## Experimental contents

### Chemicals and reagents

Methanol, acetonitrile and formic acid were HPLC grade and purchased from Fisher Co. (Pittsburg, PA, USA). Purified water was prepared by a Milli-Q ultrapure water system (Millipore, Bedford, MA, USA). Other chemicals were analytical grade. The dry roots of Raw PM (*Polygonum multiflorum* Thunb.) were purchased from Sichuan Qinbashan Chinese Herbal Medicines Pieces Ltd. (Bazhong City, Sichuan province, China) and authenticated by Prof. Jin Pei (The section of pharmacognosy, Chengdu University of traditional Chinese medicine, China).

### Animals

48 SPF Sprague-Dawley rats (200 ± 20 g) were obtained from the Laboratory Animal Center of Sichuan Academy of Traditional Chinese Medicine (Certification number SCXK 2013-19). All animals were acclimated for 7 days before the experiment. They were housed in an environmentally controlled room at a constant temperature (22 ± 2°C) and humidity (50 ± 2%) on a 12 h light/dark cycle and provided with standard diet and water. This study was carried out in accordance with the recommendations of “the Guide for the Care and Use of Laboratory Animals (NIH publication #85-23, revised in 1985) and the Guiding Principles for the Care and Use of Laboratory Animals of China.” And the experiment involving biohazards, biological agents and reagents was carried out under “the guidance of safety procedure of Chengdu University of Traditional Chinese Medicine.” The protocol was approved by “the ethical committee of Chengdu University of Traditional Chinese Medicine (No. 20160705).”

### Preparation of drugs

The plant quality was important in the toxicity evaluation. In previous experiment, fingerprint analysis of PM has been carried out in 25 batches of raw PM samples purchased from different provinces of China to compared the herb quality of different sources. Raw PM from Dabashan, Sichuan Province was chosen for toxicity evaluation (Li et al., 2017). The contents of 16 chemical compounds including gallic acid, procyanidinsB1, catechin, gallate, aloe emodin-8-β-o-glucoside, polydatin, stilbene glycosides, rhaponticin, resveratrol, emodin-8-β-o-glucoside, physcion-8-β-o-glucoside, aloe emodin, rhein, emodin, chrysophanol, and physcion were 107.43, 106.45, 851.87, 287.30, 108.62, 277.66, 43,982.58, 1,180.41, 311.32, 3,234.45, 463.20, 59.93, 35.86, 1,287.06, 4.58, 656.47 μg/g, respectively.

Raw PM was soaked for 2 h with 10 times of water, and then boiled for two times (2, 1.5 h respectively). The filtrate was collected after extraction and concentrated by rotary evaporators at 60°C. The final 2 g crude PM/mL was stored at 4°C for experiment.

### Drug administration

According to Chinese Pharmacopoeia, 3~6 g raw PM was suggested for human clinical application. After transformation from human to rat, the clinical dosage for rat was 0.3125~0.625 g/kg. In the long term toxicity experiment, it was usually recommended that 10–30 times of the clinical dosage was used as the low dose, 30–50 times as the middle dose, 50 and 100 times as the high dose. Considering the requirement and the actual administration volume, 10, 20, 40 g/kg were chosen as the low, middle and high dosage in this experiment, respectively.

The animals were randomly divided into four groups, including control group, raw PM high dose group (PMR-H), raw PM middle dose group (PMR-M) and raw PM low dose group (PMR-L). PMR-H, PMR-M and PMR-L was orally administered of 40.0, 20.0, 10.0 g/kg raw PM once per day for 28 days, respectively. Control group was orally given an equivalent volume of distilled water. The sign, behavior, urine, defecation and secretions were observed every day. The body weight was recorded on the 1st, 4th, 7th, 10th, 13th, 16th, 19th, 22nd, 25th, and 28th day, and the body weight growth rate was calculated.

### Samples collection

Twelve hour after last administration, the blood samples were collected from the rat abdominal aorta and incubated at 37°C for 30 min. Then the blood samples were centrifuged at 3,500 g for 10 min and the supernatant serum was transferred into clean plastic tube for blood biochemical analysis and metabolomics analysis. The livers were removed from the rats immediately after sacrifice.

### Sample analysis

#### Serum biochemistry and histopathological analysis

According to the kit instruction, the levels of aspartate aminotransferase (AST), alanine aminotransferase (ALT), alkaline phosphatase (ALP), total protein (TP), total bilirubin (T-BIL), direct bilirubin (D-BIL), total cholesterol (TC), triglycerides (TG) and total bile acid (TBA) were measured by Mindray BS-300 automatic biochemistry Analyzer (Shanghai Fuzhong Biosciences Corporation, China). For histopathological examination, liver tissues were fixed and preserved in 10% neutral formalin, and embedded in paraffin after dehydration. The slices (5 mm) were observed after being stained with hematoxylin and eosin (HE).

#### Metabolomics study of serum samples

Three hundred microliter serum samples were mixed with 600 μL methanol for 1 min. The samples were collected after being centrifuged at 12,000 rpm at 4°C for 10 min and the supernatant was transferred to centrifuge tube and filtered through microporous membrane (0.22 μm). The quality control (QC) sample was prepared in an equal volume of all samples, and QC sample was detected every 10 samples throughout the injection.

Metabolomics analysis was performed on Agilent 1290 UPLC-6540 Q-TOF MS/MS (Agilent Technologies, Palo Alto, USA) system which was equipped with a binary solvent delivery and an auto sampler. Analysis was carried out on an UPLC BEH C_18_ column (1.7 μm, 2.1 × 100 mm) at 40°C. The mobile phase was a mixture of solvent A (Water with 0.1% formic acid) and solvent B (Acetonitrile with 0.1% formic acid). The gradient elution was optimized as following: 0~0.5 min, B (2%); 0.5~9 min, B (2% ~50%); 9.0~12.0 min, B (50%~98%); 12.0~13.0 min, B (98%), 13.0~14.0 min, B (98%~2%), 14.0~15.0 min, B (2%). The flow rate was set at 0.30 mL/min. 8 μL supernatant were injected for UPLC-Q-TOF-MS analysis.

MS condition: Electrospray voltage was 5.5 kV in positive ionization mode and 4 kV in negative ionization mode. Declustering potential was 80 V and the source temperature was 600°C. The curtain gas was 35 psi and the nebulizer was 45 pisg for positive and 35 pisg for negative. The sheath gas flow was 12 L/min at 350°C. Calibrations were automatically conducted from m/z 50–1,000 with sodium formate solution.

### Data processing and pattern recognition analysis

#### Data pre-processing

UPLC-MS data was converted into cdf format by Protein Wizard software and XCMS (www.bioconductor.org/) was used to extract the peak data, peak denoise, peak matching, peak alignment, export and save the results for non-target metabolomics. In each sample, peak areas of metabolites were normalized by sum method applied in metaboanalyst (www.metaboanalyst.ca). A data set of all samples, consisting of retention time and normalized peak area of metabolites, was imported into Simca-P software (version 13.0, Umetrics AB, Umea, Sweden) for data pre-processing. The data were processed by unit variance scaling and mean-centered method, followed by multivariate analysis, including principal component analysis (PCA) and partial least squares-discriminant analysis (PLS-DA). Permutation test was used to prevent overfitting of the OPLS-DA model. Variables with importance parameters value higher than 1 (VIP > 1.0) in OPLS-DA model were selected as potential variables. SPSS 21.0 (Chicago, IL, USA) software was used to perform Student's *t*-test and One-way analysis of variance (ANOVA) for these variables. Metabolites with *p* < 0.05 (ANOVA and *t*-test) and fold change value >1.5 were considered to be statistically significant.

#### Biomarker identification and metabolic pathway analysis

The significant metabolic biomarkers were screened using the exact molecular ion measured by MS/MS. The potential biomarkers were tentatively identified by matching the accurate mass charge ratio and MS/MS fragmentation patterns to the online database (20 ppm as the accepted mass error) such as METLIN database (http://www.metlin.scipps.edu/) and HMDB database (http://www.hmdb.ca/). The pathways of potential biomarkers was analyzed using MetaboAnalyst 3.0 (http://www.metaboanalyst.ca/). Rattus norvegicus (rat) (81) for pathway path library was selected for pathway enrichment and topological analysis, and other parameters were set at default.

## Results

### Behavioral observations

**Figure 1:**
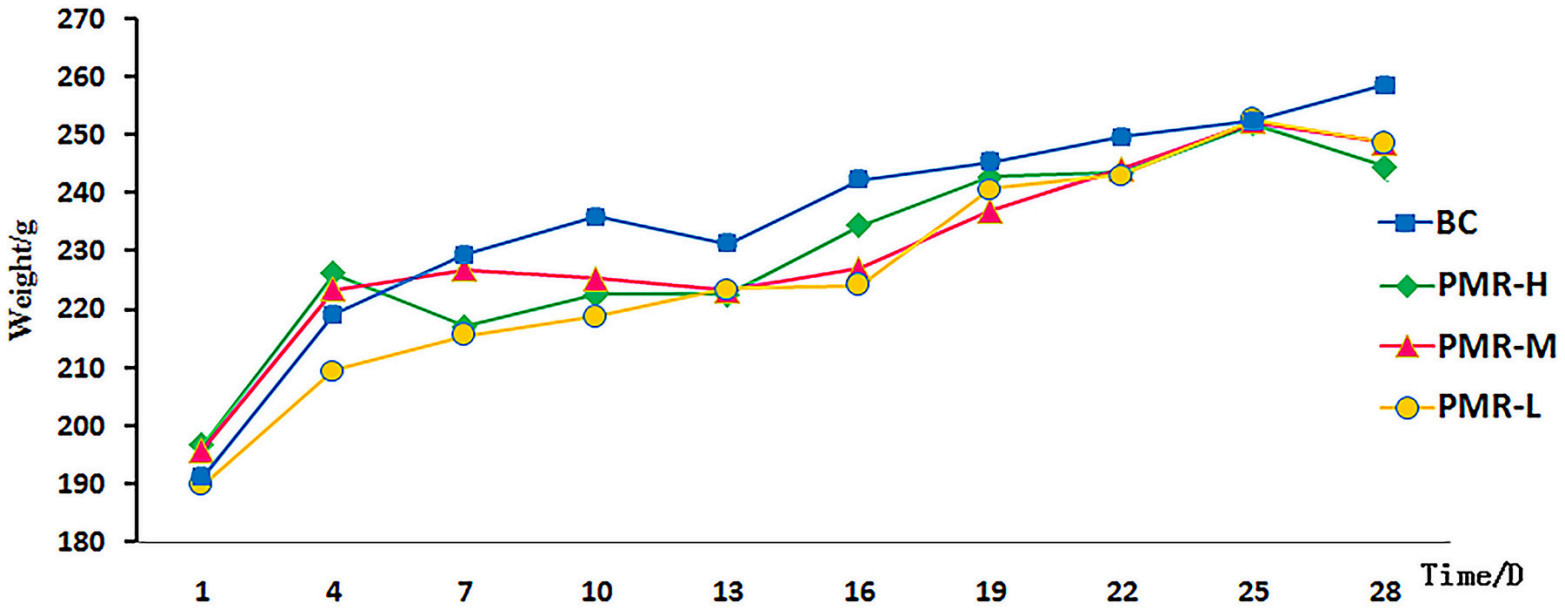
Body weight of rats in each groups in 28 days.

The rats in control and PWR-L groups had normal mental activity, smooth body hair, normal urine and defecation. However, the behavioral activity of rats decreased with yellow hair and filthy crissum in PWR-M and PWR-H groups. The body weight of each group increased with time, and no significant difference was observed among groups during study period (Figure 1). Table 1 showed that the liver of rats in PWR-M and PWR-H groups had a certain degree of swelling and the organ coefficients (calculated as Equation 1) were significantly higher than that of the control group. As a commonly used toxicology indicator, organ coefficient reflected the liver toxicity of middle and high doses of Raw PM.

(1) $$\begin{array}{r} {\text{Organ coefficient }=\text{ organ weight}/\text{rat weight}}^{*}100\% \end{array}$$

**Table 1:** The organ weight and organ coefficient of liver in each group ($\bar{x}\pm s$, *n* = 12).

**Group**	**W/g**	**Liver**	
		**W/g**	**Coe%**
BC	258.68 ± 30.04	8.18 ± 1.20	3.16 ± 0.48
PMR-L	248.65 ± 24.75	8.04 ± 1.00	3.23 ± 0.40
PMR-M	248.75 ± 50.15	9.89 ± 1.51	3.98 ± 0.57^*^
PMR-H	244.64 ± 33.53	10.41 ± 1.57	4.25 ± 0.65^**^

*Compared with the control group*,

* *p < 0.05*,

** *p < 0.01. W, weight; Coe, organ coefficient*.

### Biochemical analysis and histopathological observations

**Figure 2:**
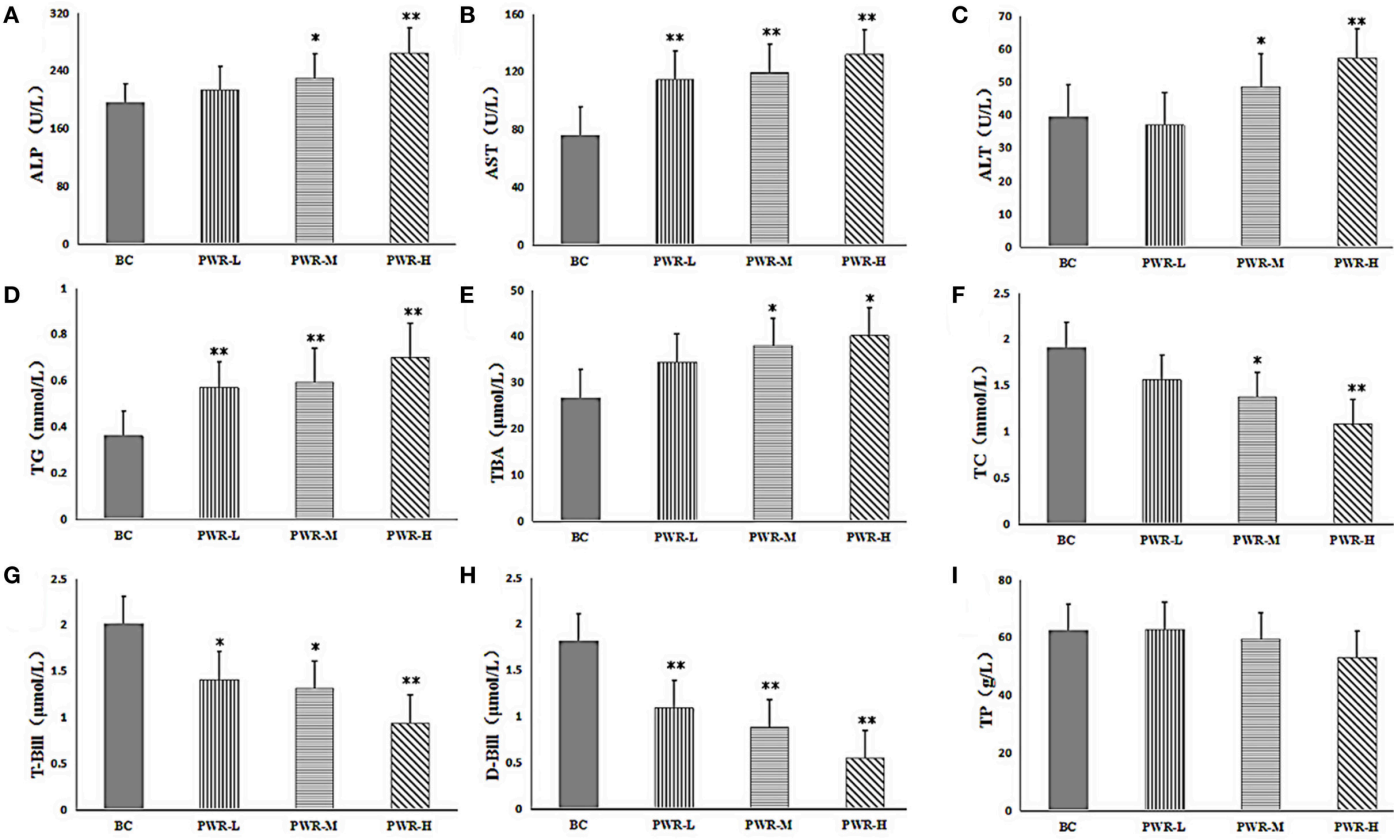
**(A–I)** The serum levels of ALP, AST, ALT, TG, TBA, TC, T-BIL, D-BIL, and TP. Compared to the control group, ^*^P < 0.05, ^**^P < 0.01. One-way analysis of variance (ANOVA) was used to calculate significant difference.

The results of biochemical analysis were shown in Table 2 and Figures 2A–I. Compared with the control group, the contents of ALT, AST, ALP, TG and TBA in PMR-M and PMR-H group were significantly increased (*P* < 0.05 or *P* < 0.01). Meanwhile, the contents of T-Bil, D-Bil, TC, and TP in serum were significantly decreased (*P* < 0.05 or *P* < 0.01). However, except serum AST, TG, T-Bil and D-Bil, no significant differences were observed between PMR-L group and the control group.

**Table 2:** The results of biochemical analysis in rats of liver injury ($\bar{x}\pm s$, *n* = 12).

**Index**	**BC**	**PMR-H**	**PMR-M**	**PMR-L**
ALP (U/L)	195.73 ± 46.23	264.48 ± 80.97^**^	229.05 ± 66.04^*^	213.87 ± 82.42
AST (U/L)	75.95 ± 22.98	131.80 ± 21.84^**^	119.20 ± 18.48^**^	114.36 ± 21.11^**^
ALT (U/L)	39.24 ± 7.58	57.23 ± 48.70^**^	48.48 ± 8.32^*^	36.90 ± 5.44
TG (mmol/L)	0.36 ± 0.39	0.70 ± 0.54^**^	0.59 ± 0.25^**^	0.57 ± 0.25^**^
TBA (μmol/L)	26.74 ± 6.67	40.13 ± 4.99^*^	37.98 ± 7.08^*^	34.44 ± 5.46
TC (mmol/L)	1.91 ± 0.22	1.08 ± 0.30^**^	1.37 ± 0.27^*^	1.56 ± 0.25
T-Bill (μmol/L)	2.01 ± 0.30	0.94 ± 0.53^**^	1.31 ± 0.40^*^	1.41 ± 0.44^*^
D-Bill (μmol/L)	1.81 ± 0.26	0.55 ± 0.30^**^	0.88 ± 0.29^**^	1.09 ± 0.43^**^
TP (g/L)	62.36 ± 2.98	52.88 ± 3.98	59.33 ± 2.66	62.75 ± 3.58

*Compared with the control group*,

* *p < 0.05*,

** *p < 0.01*.

**Figure 3:**
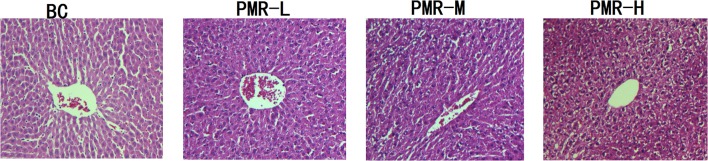
Representative light photomicrographs of rat liver specimens for HE analysis (400^*^ magnification).

After hematoxylin-eosin (HE) staining, the structure of normal liver cells observed under microscopy (Figure 3) showed that the uniform liver cells were cord-like arranged with the clearly visible nucleus and nuclear membrane. After administration of Raw PM, cell shrinking appeared with disordered cord arrangement. Karyopyknosis was detected with blurred structure, and the edge was unclear. Combined the results of biochemical analysis and pathology analysis, the liver function lesion was found in rats after Raw PM administration. Severer hepatocellular cytopathic effect was found with the increased dose.

### Multivariate statistical analysis and potential biomarkers

Serum chromatography was obtained by UPLC-Q-TOF-MS in both positive ion and negative ion mode. To obtain the difference of metabolic components among the four group samples, the multivariate statistical analysis method was used to investigate the serum metabolites of each sample.

**Figure 4:**
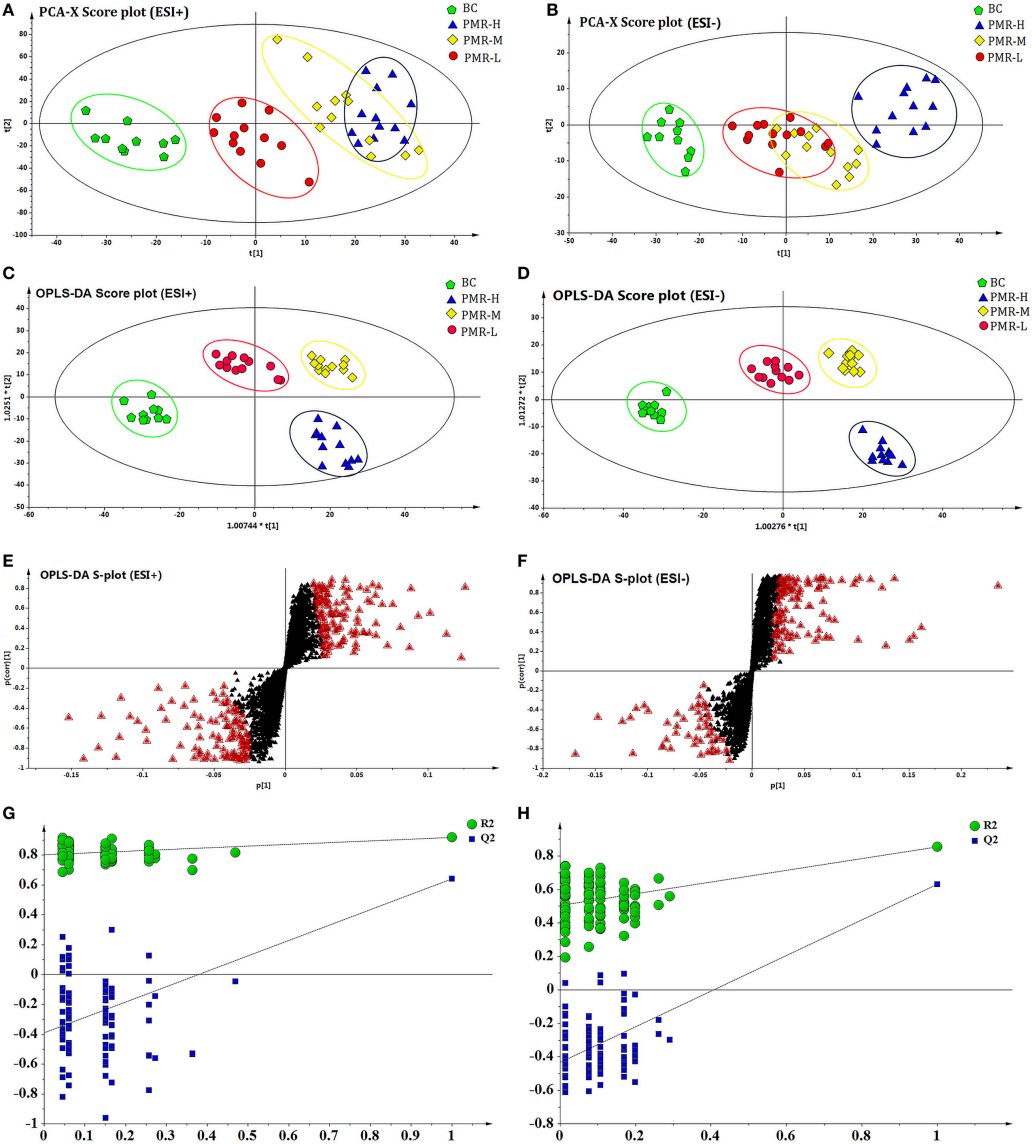
PCA score plots and OPLS-DA score plots of different groups of PM in ESI^+^ and ESI^−^ mode respectively. The score plots of PCA of BC, RPM-L, PMR-M, and PMR-H groups from PCA in ESI^+^ **(A)** and ESI^−^ **(B)** for BC group vs. PMR-L, PMR-M, PMR-H groups. The score plots of OPLS-DA of BC, PMR-L, PMR-M, and PMR-H groups from PCA in ESI^+^ **(C)** and ESI^−^ **(D)** for BC group vs. PMR-L, PMR-M, PMR-H groups. Metabolites with VIP > 1 were marked with a red triangle in ESI^+^ **(E)** and ESI^−^ **(F)**. 200-permutation test of PLS-DA model in ESI^+^ **(G)** and ESI^−^ **(H)**.

After the peak matching, all the variables of the control group and PMR groups were analyzed by PCA. An anomaly sample was removed in the clustering of the data and the result score plots of positive and negative modes were shown in Figures 4A,B. As the picture showed, an obvious separation trend can be observed among the control and three PMR groups in both positive and negative models. Although a part coincide can be observed between the PMR-M and PMR-H group or PMR-M and PMR-L groups. The separation between PMR-H and PMR-L was achieved completely. The results suggested that there were a considerable metabolite difference between the control group and drug administration groups. Significant metabolic differences between the PMR-H and PMR-L group indicated that further multivariate statistical analysis was necessary to discern the relationship among these three administration dosage.

As a supervised method, OPLS-DA was applied to further confirm the potential biomarkers of liver damage caused by PM. The results of OPLS-DA model derived from data of ESI^+^ and ESI^−^ analysis was displayed in Figures 4C–H. Figures 4C,D showed obvious separation among four groups. The samples of control group clustered together and remained relatively far from other three PMR groups. Among the three PMR groups, PMR-L and PMR-M groups separated with relatively closer distance. While the samples of PMR-H group fell far apart and aggregated in another region. The parameter R^2^Y and Q^2^ obtained by cross-validation were 0.912 and 0.625 in positive mode, 0.888 and 0.647 in negative mode (Figures 4G,H), which indicated the good predictive ability of how well the model fit the data. Loading plot with Figures 4E,F suggested the potential metabolites with variable influence on the projection (VIP > 1) which were flagged with a red triangle. Permutation test in Figures 4G,H was performed using 200 iterations in order to prevent over-fitting of the OPLS-DA model, and the results showed that the model was not over-fitted.

### The selection, identification, and analysis of potential biomarkers

**Figure 5:**
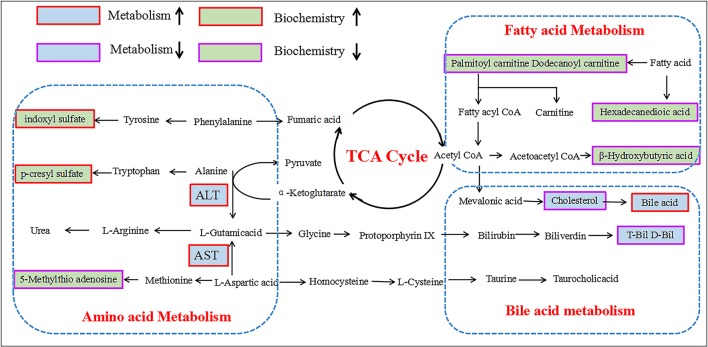
Schematic diagram of the metabolic pathway related to PM induced rat liver injury. The boxes bordered in red and purple represent metabolites that are significantly higher and lower in the PM groups than in BC group, respectively. The light green and light blue boxes indicate metabolites significantly higher and lower in the model group than in the control group, respectively. The related metabolic pathways are cycled in a black box; ALT, glutamate pyruvate transaminase; AST, aspartate aminotransferase.

**Figure 6:**
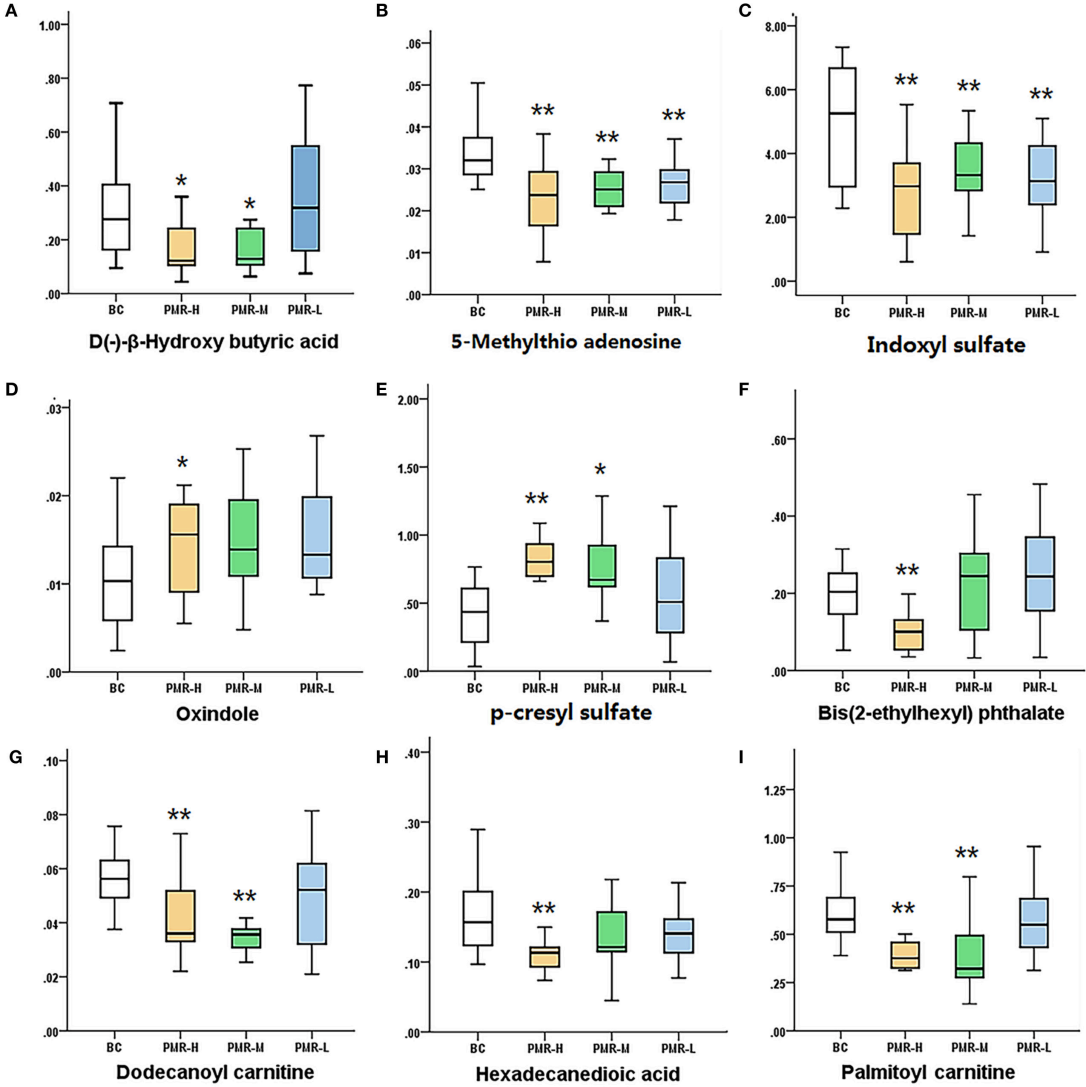
Variations in the trends of the metabolites that are biomarkers. **(A–I)** shown are the variations in the trends of D(–)-β-hydroxy butyric acid, 5-methylthio adenosine, indoxyl sulfate, oxindole, *p*-Tolyl sulfate,bis(2-ethylhexyl) phthalate, dodecanoyl carnitine, hexadecanedioic acid, palmitoyl carnitine, respectively. ^*^P < 0.05, ^**^P < 0.01 compared with the control group, respectively.

According to variable importance of the project (VIP), the differentiated metabolites with VIP value > 1 from the OPLS-DA score plots was selected for further *T*-test and ANOVA analysis using SPSS 21.0 software. Non-parametric tests were performed to statistically identify significant differences in metabolites with *P* < 0.05 and 1.5-fold-change > 1.5 between the control group and three PMR groups. Through the above two steps, differentiated metabolites related to liver injury of Raw PM were screened out. Based on the accurate mass number, retention time, MS/MS information of the metabolites, the metabolite ions were identified by the combination of online database information and standard sample spectra. Combined with the accurate mass-to-charge ratio in METLIN database and HMDB database, a total of 9 metabolites in positive and negative ion mode had been differentially expressed between in control and PMR groups. The results were summarized in Table 3 and the relative concentrations of 9 metabolites were shown in Figure 5. The important plasma biomarkers related to liver injury were further analyzed and the possible metabolic pathway of PM-induced toxicity was displayed in Figure 6.

**Table 3:** List of the identification of potential biomarkers among groups.

**No**.	**rt/min**	**Mass**	**Type**	**ppm**	**Identified compound**	**Formula**
1	2.03	104.0473	[M-H]^−^	5	D(–)-β-hydroxy butyric acid	C_4_H_8_O_3_
2	3.91	297.0849	[M+H]^+^	1	5-Methylthio adenosine	C_11_H_15_N_5_O_3_S
3	4.57	213.0096	[M-H]^−^	0	Indoxyl sulfate	C_8_H_7_NO_4_S
4	4.65	133.0528	[M+H]^+^	3	Oxindole	C_8_H_7_NO
5	5.98	188.0143	[M-H]^−^	0	p-Cresyl sulfate	C_7_H_8_O_4_S
6	10.02	390.277	[M+H]^+^	7	Bis(2-ethylhexyl) phthalate	C_24_H_38_O_4_
7	10.44	343.2722	[M+H]^+^	1	Dodecanoyl carnitine	C_19_H_37_NO_4_
8	11.5	286.2144	[M-H]^−^	0	Hexadecanedioic acid	C_16_H_30_O_4_
9	11.55	399.3349	[M+H]^+^	1	Palmitoyl carnitine	C_23_H_45_NO_4_

## Discussion

### Biochemical analysis and histopathological change

The changes of serum ALT, AST, and ALP reflected the damage degree of liver cells (Franson, 1982; Penndorf et al., 2013; Kunutsor et al., 2014). The levels of rat serum ALT, AST, and ALP exposed to middle and high dose of Raw PW were significantly increased, which indicated that the liver cell membrane was impaired and the release of them into blood was increased after PMR administration. Indirect bilirubin was taken and metabolized to D-Bil by liver cells. The function impairment of metabolic synthesis and bile secretion caused by liver damage resulted in the decreased D-Bill content. The content of T-Bill, the combination of indirect bilirubin and D-Bill, may be corresponding reduced with the decrease D-Bill content. The level of TBA reflected the function of liver synthesis and reabsorption (Henkel et al., 2013; Ferslew et al., 2015). When the liver was damaged, it cannot effectively re-intake TBA for enterohepatic circulation, which caused the increased TBA level in blood. TP directly reflected the anabolic function of liver cells (Tanaka et al., 1992; Gao et al., 2014). The decreased blood TP had a positive correlation to the degree of liver synthesis capacity dysfunction. Serum TG and TC levels also responded to the energy metabolism of the liver function (Frajacomo et al., 2012; Chen et al., 2014). The biochemical results showed that PMR could damage the liver by affecting the synthesis, transformation, secretion and energy metabolism of the liver.

### Lipid metabolism, amino acid metabolism, and bile acid metabolism involved in PMR liver toxicity

In previous report, the mechanism of hepatotoxicity induced by PM has been investigated related to the determination of biochemical markers and the analysis of metabolomics (Wang et al., 2012; Xia et al., 2017). The hepatitis biochemical results showed AST, ALT, glutamyl transpeptidase (GGT), TBA increase, and superoxide dismutase (SOD) and TBIL significant reduction. The biochemical results of our experiment were consistent with the results of these literatures. The mechanism of hepatotoxicity reported mainly includes: bile acid metabolism (Dong et al., 2015), sphingolipid metabolism and tricarboxylic acid cycle (Li et al., 2016), vitamin B6 metabolism and tryptophan metabolism (Zhang et al., 2016a). In this study, non-targeted metabolomics was used to analyze the liver toxicity of PM decoction, and the results showed that fatty acid metabolism, amino acid metabolism and bile acid metabolism were involved in the toxicity mechanism.

#### Metabolites alteration related to fatty metabolism in raw PM liver toxicity

As a necessary nutrient for the body, free carnitine played a vital role in energy production and fatty acid metabolism. Under normal circumstances, intracellular long-chain fatty acid formed and transformed into fatty acyl CoA under the action of the ester of acyl CoA synthetase which was located in the endoplasmic reticulum and mitochondrial outer membrane (Gao et al., 2013; Paczkowski et al., 2014; Meadows and Wargo, 2015). To complete β-oxidation, the fatty acyl CoA had to be combined with L-carnitine to form carnityl carnitine to enter mitochondria under the action of carnitine acyltransferase I (CPT-I), and then later released L-camitine by carnitine acyltransferase (CPT-II). Then fatty acyl CoA in mitochondria was β-oxidized to form acetyl-CoA (Prip-Buus et al., 1992; Fulgencio et al., 1996; Skrede et al., 1997). In this study, the significantly reduced long-chain acyl carnitine may be caused by the impaired activity of fatty acid esterase or CPTI which was the results of PM induced liver damage. Due to the decrease of long chain acyl carnitine, the content of fatty acids that can be oxidized and decomposed was reduced. Corresponding, the formation of the intermediate product of ketone bodies oxidative decomposition, such as acetoacetic acid, β-hydroxybutyric acid and acetone was reduced.

#### Metabolites alteration related to bile acid metabolism in raw PM liver toxicity

As the intermediate product of glycometabolism, lipid metabolism and protein metabolism, acetyl CoA was involved in the synthesis of total cholesterol under the action of hydroxymethylglutarate CoA reductase (HMG-CoA). Total cholesterol was catalyzed by 7A-hydroxylase to produce bile acid (TBA) which entered the liver and gut circulation (Parker et al., 2013; Jiang et al., 2015). The liver was injured and cannot effectively retake TBA in the enterohepatic circulation, leading to increased blood TBA content, which decreased cholesterol content by inhibiting cholesterol formation through the body feedback regulation. The damaged liver resulted in the weakened capacity of hepatocytes uptake, binding and secretion (Luo et al., 2014; Masubuchi et al., 2016). Also, the reduced uptake of free bilirubin (B-Bil) by liver cell resulted in the decreased binding of B-Bill and albumin to produce direct bilirubin (D-Bil) (Liu et al., 2012). Therefore, the amount of D-Bill discharged into the blood through the bile acids was reduced.

#### Metabolites alteration related to amino acid metabolism raw PM liver toxicity

The metabolite sulfuric acid p-cresol, produced from the amino acid phenylalanine and tyrosine by intestinal anaerobic bacteria, and the metabolite indole phenol sulfate, produced from the tryptophan decomposition products indole, were the representative substances of uremic toxins. Both sulfuric acid p-cresol and indole phenol sulfate had vascular toxicity and nephrotoxicity (Raff et al., 2008; Gironès et al., 2014). Sulfated acid p-cresol caused stress response in renal tubular cells, stromal cells, vascular smooth muscle cells through the induction of systemic oxidative stress reaction, and inhibiting endothelial proliferation and repair which led to chronic kidney disease (Poveda et al., 2014). Indole phenol sulfate promoted renal interstitial mononuclear/macrophage infiltration, produced a variety of fibrosis factors and induced renal interstitial fibrosis (Lin C. J. et al., 2015). The increased levels of the two components indicated that Raw PM not only damaged the liver, but also injured the kidneys in a certain degree.

*In vivo*, Amino acid was partly deaminized and entered blood to form blood ammonia. In the liver, arginine was hydrolyzed through the ornithine cycle to produce urea which was excreted by the kidney. Part of Amino acid was transformed into ionic ammonia by the kidney and excreted with the urine. Glutamine was produced by amino acid and glutamic acid which was originated from α-ketoglutaric acid by ALT and AST enzyme catalyzation in tricarboxylic acid cycle (Sözen et al., 2011; Miles et al., 2015). The long term administration of Raw PM resulted in liver damage with the decreased function of transforming blood ammonia to urea. And the accumulation of blood ammonia aggravated the degree of liver injury (Daijo et al., 2017; Kido et al., 2017). Then the formation of glutathione precursor adenosine methionine (SAM) was reduced, and the level of its metabolite, 5-methylthio adenosine MTA, was corresponding reduced.

## Conclusion

This study systematically carried out the toxicity investigation of PMR based on biochemical analysis, histopathological observation and metabolomics study. The related biomarkers of liver injury induced by Raw PM were screened based on metabolomics study. Pathway analysis showed that the liver damage was induced through amino acid metabolism, lipid metabolism and bile acid metabolism disorder which were in accordance with results of biochemical analysis and histopathological observations. The results showed that there was a certain correlation between the liver injury and the dose of PM. In the tested dosage range, 30 times of clinical dosage (10 g/kg for rat in the experiment) caused little liver toxicity which suggested the safety of Raw PM with normal dosage. But the potential hepatic and renal toxicity occurred with 20 and 40 g/kg also warned us not to take higher dosage of PM for a long time.

## Author contributions

Y-XL and X-HG conducted the experiments, wrote the manuscript and prepared figures. X-HG and M-CL conducted sample collection and data analysis. CP, PL, and Y-TW conceived the study.

### Conflict of interest statement

The authors declare that the research was conducted in the absence of any commercial or financial relationships that could be construed as a potential conflict of interest.

## References

[B1] Bose U. Hewavitharana A. K. Vidgen M. E. Ng Y. K. Shaw P. N. Fuerst J. A. (2014). Discovering the recondite secondary metabolome spectrum of Salinispora species: a study of inter-species diversity. PLoS ONE 12:e91488 10.1371/journal.pone.0091488 PMC3951395 24621594

[B2] Cárdenas A. Restrepo J. C. Sierra F. Correa G. (2006). Acute hepatitis dueto shen-min: a herbal product derived from *Polygonum multiflorum*. J. Clin. Gastroenterol. 40, 629–632. 10.1097/00004836-200608000-00014 16917407

[B3] Chen J. W. Liu D. Xue Z. Y. Chen B. Zhou Y. Chen S. R. (2014). Research on therapeutic effect of yangganjiejiu prescription on alcoholic fatty liver and its mechanism. Zhong Yao Cai 37, 293–298. 10.13863/j.issn1001-4454.2014.02.034 25095354

[B4] Cho H. C. Min H. J. Ha C. Y. Kim H. J. Kim T. H. Jung W. T.. (2009). Reactivation of pulmonary tuberculosis in a patient with *Polygonum multiflorum* Thunb-induced hepatitis. Gut liver 3, 52–56. 10.5009/gnl.2009.3.1.52 20479902 PMC2871557

[B5] Daijo K. Kawaoka T. Nakahara T. Nagaoki Y. Tsuge M. Hiramatsu A.. (2017). Late-onset ornithine transcarbamylase deficiency associated with hyperammonemia. Clin. J. Gastroenterol. 10, 383–387. 10.1007/s12328-017-0753-0 28597413

[B6] Dong H. Slain D. Cheng J. Ma W. Liang W. (2014). Eighteen cases of liver injury following ingestion of *Polygonum multiflorum*. Complement. Ther. Med. 22, 70–74. 10.1016/j.ctim.2013.12.008 24559819

[B7] Dong Q. Li N. Li Q. Zhang C. E. Feng W. W. Li G. Q.. (2015). Screening for biomarkers of liver injury induced by *Polygonum multiflorum*: a targeted metabolomic study. Front. Pharmacol. 6:217. 10.3389/fphar.2015.00217 26483689 PMC4591842

[B8] Ferslew B. C. Xie G. Johnston C. K. Su M. Stewart P. W. Jia W.. (2015). Altered bile acid metabolome in patients with nonalcoholic steatohepatitis. Dig. Dis. Sci. 60, 3318–3328. 10.1007/s10620-015-3776-8 26138654 PMC4864493

[B9] Frajacomo F. T. Demarzo M. M. Fernandes C. R. Martinello F. Bachur J. A. Uyemura S. A.. (2012). The effects of high-intensity resistance exercise on the blood lipid profile and liver function in hypercholesterolemic hamsters. Appl. Physiol. Nutr. Metab. 37, 448–454. 10.1139/h2012-008 22494106

[B10] Franson J. C. (1982). Enzyme activities in plasma, liver and kidney of black ducks and mallards. J. Wildl. Dis. 18, 481–485. 10.7589/0090-3558-18.4.481 6130168

[B11] Fulgencio J. P. Kohl C. Girard J. Pégorier J. P. (1996). Troglitazone inhibits fatty acid oxidation and esterification, and gluconeogenesis in isolated hepatocytes from starved rats. Diabetes 45, 1556–1562. 10.2337/diab.45.11.1556 8866561

[B12] Gao F. Liu Y. Li L. Li M. Zhang C. Ao C.. (2014). Effects of maternal undernutrition during late pregnancy on the development and function of ovine fetal liver. Anim. Reprod. Sci. 147, 99–105. 10.1016/j.anireprosci.2014.04.012 24852270

[B13] Gao S. Casals N. Keung W. Moran T. H. Lopaschuk G. D. (2013). Differential effects of central ghrelin on fatty acid metabolism in hypothalamic ventral medial and arcuate nuclei. Physiol. Behav. 118, 165–170. 10.1016/j.physbeh.2013.03.030 23680429

[B14] Gironès N. Carbajosa S. Guerrero N. A. Poveda C. Chillón-Marinas C. Fresno M. (2014). Global metabolomic profiling of acute myocarditis caused by *Trypanosoma cruzi* infection. PLoS Negl. Trop. Dis. 8:e3337. 10.1371/journal.pntd.0003337 25412247 PMC4239010

[B15] Henkel A. S. Gooijert K. E. Havinga R. Boverhof R. Green R. M. Verkade H. J. (2013). Hepatic overexpression of Abcb11 in mice promotes the conservation of bile acids within the enterohepatic circulation. Am. J. Physiol. Gastrointest. Liver Physiol. 304, 221–226. 10.1152/ajpgi.00322.2012 23139217 PMC3543647

[B16] Jiang C. Wang Q. Wei Y. Yao N. Wu Z. Ma Y.. (2015). Cholesterol-lowering effects and potential mechanisms of different polar extracts from *Cyclocarya paliurus* leave in hyperlipidemic mice. J. Ethnopharmacol. 176, 17–26. 10.1016/j.jep.2015.10.006 26477373

[B17] Kido J. Kawasaki T. Mitsubuchi H. Kamohara H. Ohba T. Matsumoto S. (2017). Hyperammonemia crisis following parturition in a female patient with ornithinetranscarbamylase deficiency. World J. Hepatol. 9, 343–348. 10.4254/wjh.v9.i6.343 28293384 PMC5332424

[B18] Kunutsor S. K. Apekey T. A. Khan H. (2014). Liver enzymes and risk of cardiovascular disease in the general population: a meta-analysis of prospective cohort studies. Atherosclerosis 236, 7–17. 10.1016/j.atherosclerosis.2014.06.006 24998934

[B19] Lei X. Chen J. Ren J. T. Li Y. Zhai J. B. Mu W.. (2015). Liver damage associated with *Polygonum multiflorum* Thunb.: a systematic review of case reports and case series. Evid. Based Complement. Alternat. Med. 2015:459749. 10.1155/2015/459749 25648693 PMC4306360

[B20] Li C. Y. Tu C. Gao D. Wang R. L. Zhang H. Z. Niu M. (2016). Metabolomic study on idiosyncratic liver injury induced by different extracts of*Polygonum multiflorumin* rats integrated with pattern recognition and enriched pathways analysis. Front. Pharmacol. 7:483 10.3389/fphar.2016.00483 28018221 PMC5156827

[B21] Li Z. Y. He P. Sun H. F. Qin X. M. Du G. H. (2014). (1)H NMR based metabolomic study of the antifatigue effect of Astragali Radix. Mol. Biosyst. 10, 3022–3030. 10.1039/C4MB00370E 25201073

[B22] Li Y. X. Gong X. H. Liu M. C. Peng C. Li P. Wang Y. T. (2017). A new strategy for quality evaluation and identification of representative chemical components in *Polygonum multiflorum* Thunb. Evid. Based Complement Alternat. Med. 2017:6238464. 10.1155/2017/6238464 28243311 PMC5294750

[B23] Lian X. Ke T. T. Hu A. R. (2013). Clinical analysis of 52 cases of drug-induced liver injury caused by *Polygonum multiflorum* and its preparations. Chin. Archiv. Tradit. Chin. Med. 31, 1133–1134. 10.13193/j.archtcm.2013.05.175.lianx.085 26882605

[B24] Lin C. J. Wu V. Wu P. C. Wu C. J. (2015). Meta-analysis of the associations of p-Cresyl Sulfate (PCS) and Indoxyl Sulfate (IS) with cardiovascular events and all-cause mortality in patients with chronic renal failure. PLoS ONE 10:e0132589. 10.1371/journal.pone.0132589 26173073 PMC4501756

[B25] Lin L. Ni B. Lin H. Zhang M. Li X. Yin X.. (2015). Traditional usages, botany, phytochemistry, pharmacology and toxicology of *Polygonum multiflorum* Thunb.: a review. J. Ethnopharmacol. 159, 158–183. 10.1016/j.jep.2014.11.009 25449462 PMC7127521

[B26] Liu X. D. Wu J. L. Liang J. Zhang T. Sheng Q. S. (2012). Globulin-platelet model predicts minimal fibrosis and cirrhosis in chronic hepatitis B virus infected patients. World J. Gastroenterol. 18, 2784–2792. 10.3748/wjg.v18.i22.2784 22719186 PMC3374981

[B27] Luo L. Schomaker S. Houle C. Aubrecht J. Colangelo J. L. (2014). Evaluation of serum bile acid profiles as biomarkers of liver injury in rodents. Toxicol. Sci. 137, 12–25. 10.1093/toxsci/kft221 24085190

[B28] Ma X. Chi Y. H. Niu M. Zhu Y. Zhao Y. L. Chen Z.. (2016). Metabolomics coupled with multivariate data and pathway analysis on potential biomarkers in cholestasis and intervention effect of *Paeonia lactiflora* Pall. Front. Pharmacol. 7:14. 10.3389/fphar.2016.00014 26869930 PMC4740759

[B29] Masubuchi N. Nishiya T. Imaoka M. Mizumaki K. Okazaki O. (2016). Promising toxicological biomarkers for the diagnosis of liver injury types: bile acid metabolic profiles and oxidative stress marker as screening tools in drug development. Chem. Biol. Interact. 255, 74–82. 10.1016/j.cbi.2015.09.012 26365562

[B30] Meadows J. A. Wargo M. J. (2015). Carnitine in bacterial physiology and metabolism. Microbiology 161, 1161–1174. 10.1099/mic.0.000080 25787873 PMC4635513

[B31] Miao Y. J. Yun Y. L. (2013). Two cases of drug-induced hepatitis caused by *Polygonum multiflorum*. Chin. J. Drug Appl. Monit. 10, 61–62.

[B32] Miles E. D. McBride B. W. Jia Y. Liao S. F. Boling J. A. Bridges P. J.. (2015). Glutamine synthetase and alanine transaminase expression are decreased in livers of aged vs. young beef cows and GS can be upregulated by 17β-estradiol implants. J. Anim. Sci. 93, 4500–4509. 10.2527/jas.2015-9294 26440349

[B33] Paczkowski M. Schoolcraft W. B. Krisher R. L. (2014). Fatty acid metabolism during maturation affects glucose uptake and is essential to oocyte competence. Reproduction 148, 429–439. 10.1530/REP-14-0015 25062802

[B34] Parker R. A. Garcia R. Ryan C. S. Liu X. Shipkova P. Livanov V.. (2013). Bile acid and sterol metabolism with combined HMG-CoA reductase and PCSK9 suppression. J. Lipid Res. 54, 2400–2409. 10.1194/jlr.M038331 23614904 PMC3735938

[B35] Penndorf V. Saner F. Gerken G. Canbay A. (2013). Liver parameters in intensive care medicine. Zentralbl. Chir. 138, 636–642. 10.1055/s-0031-1271601 22565500

[B36] Poveda J. Sanchez-Niño M. D. Glorieux G. Sanz A. B. Egido J. Vanholder R.. (2014). p-cresyl sulphate has pro-inflammatory and cytotoxic actions on human proximal tubular epithelial cells. Nephrol. Dial. Transplant. 29, 56–64. 10.1093/ndt/gft367 24166466

[B37] Prip-Buus C. Bouthillier-Voisin A. C. Kohl C. Demaugre F. Girard J. Pegorier J. P. (1992). Evidence for an impaired long-chain fatty acid oxidation and ketogenesis in Fao hepatoma cells. Eur. J. Biochem. 209, 291–298. 10.1111/j.1432-1033.1992.tb17288.x 1356769

[B38] Raff A. C. Meyer T. W. Hostetter T. H. (2008). New insights into uremic toxicity. Curr. Opin. Nephrol. Hypertens. 17, 560–565. 10.1097/MNH.0b013e32830f45b6 18941347

[B39] Skrede S. Sørensen H. N. Larsen L. N. Steineger H. H. Høvik K. Spydevold O. S.. (1997). Thia fatty acids, metabolism and metabolic effects. Biochim. Biophys. Acta 1344, 115–131. 10.1016/S0005-2760(96)00138-5 9030189

[B40] Sözen S. Kisakürek M. Yildiz F. Gönültaş M. Dinçel A. S. (2011). The effects of glutamine on hepatic ischemia reperfusion injury in rats. Hippokratia 15, 161–166. 22110300 PMC3209681

[B41] Su Y. W. Tan E. Zhang J. You J. L. Liu Y. Liu C.. (2014). Study on three different species tibetan medicine sea buckthorn by 1H-NMR-based metabonomics. Zhongguo Zhong Yao Za Zhi 39, 4234–4239. 10.4268/cjcmm20142129 25775800

[B42] Tanaka E. Ishikawa A. Yamamoto Y. Osada A. Tsuji K. Fukao K.. (1992). A simple useful method for the determination of hepatic function in patients with liver cirrhosis using caffeine and its three major dimethylmetabolites. Int. J. Clin. Pharmacol. Ther. Toxicol. 30, 336–341. 1428297

[B43] Teschke R. Wolff A. Frenzel C. Schulze J. (2014). Review article: herbal hepatotoxicity–an update on traditional Chinese medicine preparations. Aliment. Pharmacol. Ther. 40, 32–50. 10.1111/apt.12798 24844799

[B44] Wang T. Wang J. Jiang Z. Zhou Z. Li Y. Zhang L.. (2012). Study on hepatotoxicity of aqueous extracts of *Polygonum multiforum* in rats after 28-day oral administration-analysis on correlation of cholestasis. Zhongguo Zhong Yao Za Zhi 37, 1445–1450. 10.4268/cjcmm20121021 22860459

[B45] Wu X. Chen X. Huang Q. Fang D. Li G. Zhang G. (2012). Toxicity of raw and processed roots of *Polygonum multiflorum*. Fitoterapia 83, 469–475. 10.1016/j.fitote.2011.12.012 22210538

[B46] Xia X. H. Yuan Y. Y. Liu M. (2017). The assessment of the chronic hepatotoxicity induced by Polygoni Multiflori Radix in rats: a pilot study by using untargeted metabolomics method. J. Ethnopharmacol. 203:182. 10.1016/j.jep.2017.03.046 28365236

[B47] Zhang C. E. Niu M. Li Q. Zhao Y. L. Ma Z. J. Xiong Y.. (2016a). Urine metabolomics study on the liver injury in rats induced by raw and processed *Polygonum multiflorum* integrated with pattern recognition and pathways analysis. J. Ethnopharmacol. 194, 299–306. 10.1016/j.jep.2016.09.011 27620661

[B48] Zhang C. E. Niu M. Li R. Y. Feng W. W. Ma X. Dong Q.. (2016b). Untargeted metabolomics reveals dose-response characteristics for effect of rhubarb in a rat model of cholestasis. Front. Pharmacol. 7:85. 10.3389/fphar.2016.00085 27065293 PMC4814850

[B49] Zhang Y. Ding T. Diao T. Deng M. Chen S. (2015). Effects of *Polygonum multiflorum* on the activity of cytochrome P450 isoforms in rats. Pharmazie 70, 47–54. 10.1691/ph.2015.4693 25975098

